# The influence of organizational learning and external cooperation configuration on enterprise technological innovation: A study based on fsQCA approach

**DOI:** 10.1371/journal.pone.0271960

**Published:** 2022-08-04

**Authors:** Junyi Du, Chunyu Zhang, Qiong Wu

**Affiliations:** School of Economics and Management, Guangxi Normal University, Guilin, Guangxi, China; Swansea University, UNITED KINGDOM

## Abstract

This study explores the influence of organizational learning and external cooperation configuration on enterprise technological innovation and constructs a comprehensive theoretical framework of "organizational learning-external cooperation-technological innovation" based on the fuzzy set qualitative comparative analysis (fsQCA) method. The results show the following. (1) A single episode of organizational learning or external cooperation cannot affect the enterprise’s technological innovation, which requires the mutual linkage of the two to improve enterprise technological innovation performance. (2) The technological innovation model in which organizational learning and external cooperation interact is an effective way for enterprises to improve technological innovation performance. There are four technological innovation models that produce high technological innovation performance, namely consciousness-system synergy, consciousness-led, quasi-full, and all-around drive. (3) There are four models of non-high-tech innovation performance, which are not opposed to the technological innovation model of high-tech innovation performance. This research expands the technological innovation perspective of organizational learning and external cooperation matching, provides enterprises with effective technological innovation activities, and provides a theoretical reference and practical guidance for improving technological innovation performance.

## 1. Introduction

Knowledge plays a significant role in the process of organizational innovation. Hence, organizational learning affects the technological innovation of enterprises [1, 2]. Previous research has validated organizational learning as an effective way for organizations to improve technological innovation [3–5], and promoted enterprise innovation performance [6]. However, some scholars posit that the process and content of organizational learning are distinct, and that the technological innovation of enterprises is quite different. Enterprises are highly motivated to learn, and managers are open-minded and actively listen to employees’ opinions. If the manager’s development planning and management methods for the enterprise deviate from the employee’s development willingness, it will have an adverse impact on technological innovation. Some research falls short by taking organizational learning as a general concept without providing sufficient detail. Therefore, scholars have begun to divide organizational learning into different aspects and dimensions to analyse the impact on enterprise technological innovation. Other scholars have examined the impact of external cooperation on the technological innovation of enterprises, [7] which is an important driving force in the development of enterprise innovation. For some enterprises, internal R&D and externally sourced innovation practices affect technological innovation [8]. Within a special range, appropriate foreign cooperative R&D and enterprise technology purchases improve the enterprise’s technological innovation efficiency. However, if it exceeds the affordability of the enterprise, enterprise costs will increase and the efficiency of its technological innovation will be affected. There are differences in economic development, population density and resources between regions. Therefore, the technological innovation activities of enterprises will be affected by the regional agglomeration effect [9]. In other words, companies in different regions have different innovation capabilities. Developed regions with complete infrastructure, high-quality labour supply, and well-established value chains and supply chains have greater innovation capabilities and innovation efficiency. Previous studies have focused on the influence of cooperation on enterprises R&D efficiency and technological innovation performance [10] and on the lack of an effective combination of organizational learning and external cooperation. Therefore, this study explores the process of organizational learning and external cooperation to promote corporate technological innovation.

Technological innovation is an important factor for enterprises to maintain competitive advantage and achieve sustainable development, which affects the enterprise innovation performance. Topics such as organizational learning, external cooperation, and technological innovation performance have attracted extensive scholarly attention. However, previous studies have linked organizational learning with technological innovation performance alone or have combined external cooperation with technological innovation performance and use quantitative research methods to explore multiple regression and interaction effects between cause and effect. However, there is a lack of research that links organizational learning and external cooperation factors to explore the joint effect on technological innovation performance. The degree of learning commitment, open-mindedness, shared vision and willingness to cooperate, the type of cooperation, and the cooperation environment are different between firms, and existing research ignores the complexity between dimensions [11]. Therefore, from an overall perspective, it is particularly necessary to study the multiconditional linkage and matching relationship between organizational learning and external cooperation. This approach will contribute to improving the technological innovation capability of enterprises, overcoming the disjunction between theoretical research and practice in the technological innovation system proposed by China, and providing effective technological innovation methods and ideas for enterprise managers. Good technological innovation performance is often the result of the combination of different dimensions of organizational learning and external cooperation, and the equivalent effect of multiple paths. Therefore, from the perspective of organizational learning, this paper uses the fuzzy set qualitative comparative analysis (fsQCA) method to combine the factors of enterprises’ external cooperation. Using the idea of set theory [12] to integrate organizational learning and external cooperation, this paper divides organizational learning and external cooperation into several different dimensions, comprehensively analyses the conditions of organizational learning and external cooperation, configures them to produce high-tech innovation performance, and builds a complete knowledge theoretical framework of "organizational learning-external cooperation-technological innovation". By combining organizational learning and external cooperation, this paper proposed effective ways and strategic models to improve the performance of enterprise technological innovation, and discusses the possible paths that affect the performance of enterprise technological innovation through the combination of different dimensions.

## 2. Literature review

Organizational learning is the process by which an organization changes and adjusts itself in order to adapt to a changing environment [13]. Organizational learning plays a decisive role in the technological innovation performance of enterprises. Previous scholars have discussed the relationship between organizational learning and innovation performance [14–16]. Organizational learning theory holds that organizational learning is the correction of organizational errors and the recombination and application of organizational theory. It is a process in which organizations absorb, understand and master high-quality knowledge resources to improve organizational action efficiency. If enterprises want to maintain their competitive advantage, they must innovate. Individual and organizational learning are conducive to organizational innovation. Innovation comes from organizational learning. Exploratory learning is conducive to improving enterprises’ independent innovation ability and collaborative innovation ability [17] so as to improve enterprises’ technological innovation performance. Organizational learning is similar to a vast reservoir of knowledge [18], which produces a linkage effect and is conducive to the sustainable development of enterprises in a dynamic business environment. Therefore, organizational learning is considered an invisible resource. It improves the management ability of the organization and enhances the understanding of employees. It is a powerful means for enterprises to maintain their core competitiveness. At the same time, knowledge resources are the most important strategic resources of the enterprise [19]. Knowledge resources determine to a large extent the competitive advantage of enterprise innovation. Hence, it determines the technological innovation performance of enterprises. Moreover, there are different levels of organizational learning that lead to the complementarity and heterogeneity of knowledge resources, which are conducive to the absorptive capacity of organizational members and improve the knowledge stock and flow of members. However, organizational learning does not necessarily improve innovation performance. Moreover, in addition to focusing on innovation, organizational learning also requires standardized management of enterprises to improve the organization’s forecasting accuracy. There is a balance between internal and external learning, and this balance can result in the best innovation performance for the organization. However, most enterprises are still in the exploratory stage or the lost stage of the equilibrium point. The process of enterprise learning expands the boundaries of knowledge and improves the heterogeneity of knowledge. Therefore, enterprises should make full use of the knowledge resources obtained through organizational learning, which maintains the core competitiveness and competitive advantage and then improves the performance of technological innovation.

From the traditional organizational learning perspective, organizational learning ability is affected by organizational cooperation/competition. There is an embedded relationship between organizational learning and organizational cooperation/ competition. Organizational cooperation is conducive to knowledge sharing. Moreover, it is an important way to improve communication and job performance. Organizational learning is inseparable from the relationship between organizations. The organization’s cooperation, competition or cocompetitive relationship effects the way, method and type of organizational knowledge acquisition. The enterprise conducts organizational learning through enterprise cooperation, which drives enterprise technological innovation. It enhances the innovation ability of enterprises and effects enterprise innovation performance. Enterprise cooperation allows all parties to share resources, reduce the differentiation of products and services, and reduce the cost of innovation [20]. At the same time, external cooperation is an important means for enterprises to reduce market risks. By establishing strategic alliances, enterprises can achieve the purpose of "complementary advantages of cooperation" [21] and form unique and sustainable competitive advantages. According to the resource dependence theory, enterprises and partners will establish an exchange relationship due to the dependence on scarce resources. According to social network theory, organizational team members have multiple identities, and the more teams connected through different identities, the more conducive to knowledge transfer and personal and organizational learning. External cooperation innovation is conducive to improving the success rate of technology research and development and the company’s technological innovation capabilities. External cooperation offsets the lack of its R&D resources and capabilities, which fully recognizes the dependence on knowledge, thereby improving knowledge integration capabilities and ultimately improving technological innovation capabilities. The cooperation performance has direct or indirect effects by substitutability, imitability and mobility of resources in the process of enterprises’ external cooperation [22]. The matching degree, willingness to cooperate and resource capacity of partners affect cooperation performance. Moreover, the relational interests, shared values, and cooperation willingness of partners affect external cooperation. There are two ways for companies to open up new markets. The company obtains technical and talent support through cooperation with universities, and the company obtains market information through cooperation with customers or suppliers [23]. According to market demand, enterprises develop new products, which promote enterprise innovation and performance. In addition, enterprises have brought cross-institutional learning effects through external cooperation. After an enterprise converts the new knowledge learned into internal resources, its technological innovation capability will be greatly improved. As external cooperation and innovation with enterprises as the main body gradually become a consensus, the willingness of external cooperation and innovation is also unprecedentedly high. Although the external cooperation of enterprises can overcome the shortcomings of their own resources, they can achieve common interests and personal interests in the process of cooperation. However, based on bounded rationality and partner opportunism, firms will protect core assets and capabilities, thereby reducing the advantages of cooperation. In general, existing research explores the relationship between innovation performance from the aspects of enterprise alliance capability, standard alliance network, innovation capability, and resource integration. However, external cooperation did not meet the expected level of technological innovation. Empirical research shows that cooperation is not a necessary condition to generate innovation, but not a sufficient condition [4]. Likewise, few studies link organizational learning and external cooperation as important influencing factors of enterprise technological innovation. Instead, it is mostly based on resource-based theory, only studies the impact of a single variable on performance, and studies traditional performance rather than technological innovation performance.

To sum up, the practice of enterprise technology innovation has a long history. The previous studies are mainly based on transaction cost theory and technological innovation theory, focusing on the research on the participants of enterprise cooperation, organizational model and cooperative innovation performance through empirical analysis. Previous studies have successively confirmed the effect of organizational learning on enterprise performance and the effect of external cooperation on enterprise performance [24, 25]. However, there are few studies on the effect of organizational learning and external cooperation on the performance of technological innovation of enterprises at the same time.

**Fig 1:**
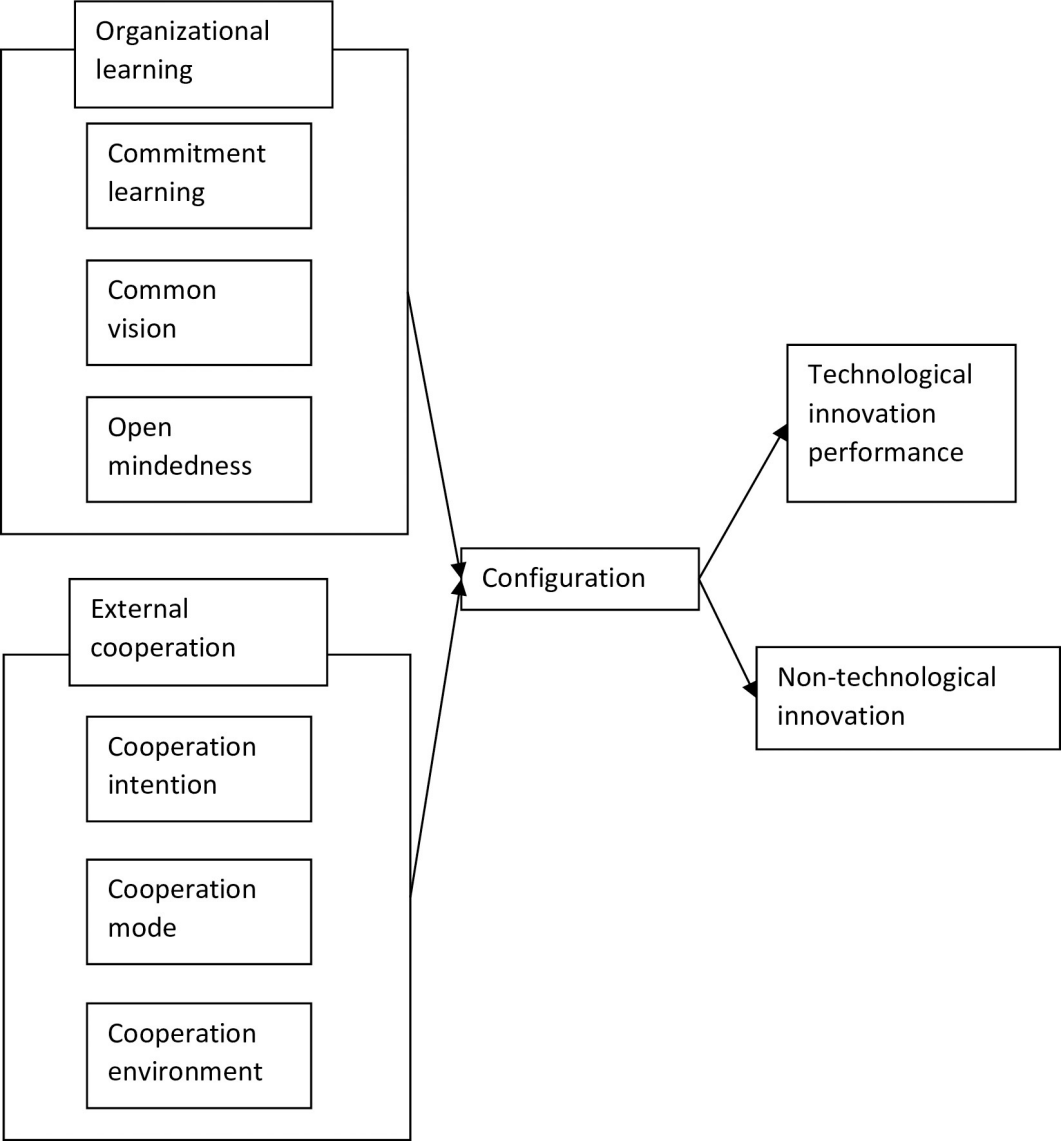
Analysis framework.

In view of this, based on organizational learning theory and social network theory, this paper explores the effect of organizational learning and external cooperation configuration on technological innovation performance (TIP). Moreover, this paper constructs a comprehensive theoretical framework of "organizational learning-external cooperation-technological innovation", and divides and matches organizational learning and external cooperation. This study refers to the research of Sinkula, Baker [26] and Hult, Ketchen Jr [27] and divides organizational learning into three components: commitment learning, common vision, and open mindedness. In addition, this paper refers to the research of Tether [28] and Tortoriello, Reagans [29], and divides external cooperation into three levels: cooperation intention, cooperation mode and cooperation environment. This paper explores the configuration mechanism that affects the enterprise technological innovation performance through six antecedent variables at the two levels of organizational learning and external cooperation. Organizational learning includes commitment learning (CL), common vision (CV), and open mindedness (OM). External cooperation includes cooperation intention (CI), cooperation mode (CM), and cooperation environment (CE), and the analytical model is shown in Fig 1.

### 2.1 The level of organizational learning

Commitment learning refers to learning as a fundamental value of a business. Commitment learning represents the willingness of enterprise members to learn. Commitment learning helps companies develop the habit of thinking dynamically, helps companies discover changes in the dynamic environment in time, and solves problems in the market and within the organization at the same time. Enterprises conduct commitment learning in a dynamic environment to capture market changes and customer needs and reduce perceived risks and opportunism. The stronger the enterprise’s sense of commitment learning, the higher the learning enthusiasm of organizational members and the lower the probability of cooperation conflict. Then, the frequency of cooperation increases, the more obvious the cooperative relationship is, and the more learning resources the enterprise has. Moreover, commitment learning strengthens the trust level and emotional connection between two or more parties [30]. The higher the commitment learning, the stronger the learning climate, the more active the communication and the wider the learning channels. Therefore, building trust and emotion with partners promotes the transfer and absorption of knowledge and improves innovation capabilities, improving innovation performance. Commitment learning has a positive impact on the integration, creation, externalization and internalization of knowledge. Commitment learning creates synergy and resource integration effects, that is, the synergistic technology learning effect of clusters, which is conducive to the deeper transfer and sharing of knowledge between enterprises and partners, thereby gaining a competitive advantage and promoting the improvement of enterprises’ technological innovation performance. The greater the commitment to learning reflects the importance of knowledge resources and the urgency of wanting to conduct organizational learning. Managers often require employees to learn actively and effectively, strive to improve the efficiency of resource integration and promote the improvement of technological innovation capabilities and performance of enterprises. Greater commitment learning is conducive to increasing the investment of enterprise members in organizational learning and improving the sensitivity to environmental changes [31], so it can accurately predict changes in business direction.

Common vision refers to the in-depth communication between the members of the organization about the company’s future development vision. It describes the common desire for the company’s development, which is conducive to improving the cohesion of the organization and promoting the realization of the organization’s goals.

The extent of the common vision represents whether the goals of the organization are consistent with those of the employees. When the goals of the members of the organization are consistent with the goals of the organization, the cohesion of the employees will be stronger. The sharing of vision prompts organizations to learn from exchanges and obtain more valuable information resources. By sharing experience and knowledge in the process of communication, it is easy to establish trust between team members, improve the efficiency and effect of knowledge interaction, and promote the spiral of knowledge innovation [32].

Open-mindedness refers to an organization that is no longer limited to traditional ideas and inertial behaviours and makes breakthroughs, and it is the embodiment of the organization’s creative learning and relearning. Due to the existence of experience inertia and learning inertia, organizations will ignore the status quo, ignore learning, and refuse to cooperate. Open minds will improve employees’ work attitudes, clarify organizational goals, innovate cultural atmosphere, and enhance personal values. An open mind can effectively avoid situations in which the organization is stagnant. Managers encourage employees to question and make reasonable suggestions, so as to carry out innovative learning and re-learning. Therefore, open mindedness expands the channels of knowledge acquisition, captures more effective information, improves the enterprise’s ability to predict the environment, and improves the efficiency of the organization in acquiring new knowledge. It has a positive catalytic effect on the transformation of explicit knowledge and tacit knowledge. Open mindedness is an important factor in knowledge creation in the learning process. It is conducive to enterprise technology research and development and improves the innovation ability of employees [33]. Therefore, it has the effect of improving the technological innovation performance of enterprises.

### 2.2 The level of external cooperation

Cooperation intention is the perception of the company and its employees that the company cooperates with other organizations. Cooperation intention is the basis for enterprises to carry out cooperative innovation activities with other organizations. It reflects the preference of enterprises to actively cooperate with other enterprises through actively seek partners, participating in external activities and sharing resources. It represents the openness of the organization. In the era of digital economy, technological innovation of enterprises needs to be realized with the help of cooperative relationships and innovation networks, and cooperation intention strengthens the enthusiasm of enterprises to carry out and cooperate in technological innovation. At the same time, external learning incurs learning costs. The stronger the cooperation intention, the greater the probability of acquiring knowledge, which can reduce the cost of organizational learning. Cooperation intention has a certain influence on creativity, and positive cooperation intention enhances the cooperation relationship between organizations, which leads to more creativity and flexibility [34]. It is conducive to improving the performance of technological innovation of enterprises.

Cooperation mode is a form of cooperation and innovation adopted by enterprises and partners. The ultimate goal of an enterprise’s external cooperation is to obtain synergies that cannot be achieved by a single enterprise, to achieve complementary resources, to form core competitiveness, and to maintain a competitive advantage. The external cooperation of enterprises can improve the R&D technology and product quality by adopting the development cooperation mode [35]. Development cooperation mainly improves customer satisfaction by improving product quality and efficiency, which is helpful for enterprises to fully understand the unpredictable potential market demand. With the advent of the digital age, companies increasingly need to improve technology, reduce R&D costs, and improve product quality and performance to meet the diverse needs of customers. The high degree of cooperation mode of the enterprise improves the enterprise products, improves the enterprise technology, and improves production efficiency, which enhances the enterprise technological innovation performance [36].

Cooperation environment refers to the external social environment in which an enterprise conducts external cooperation. It mainly analyses the cooperation environment from the aspects of government policies, laws and regulations, cooperation information platforms, and intermediaries. The government occupies a large position in the enterprise innovation system, and enterprises can more easily and actively carry out external cooperation under the environment of government funding, government guarantee and policy support. The government has the responsibility and obligation to provide an excellent cooperation environment, policy environment, legal environment and venture investment environment for enterprise cooperation. Direct government funding and tax incentives are all conducive to the technology research and development of enterprises. The more support and cooperation information an enterprise obtains from government policies, information platforms and intermediary service agencies, the greater the enterprise’s cooperation intention, and the stronger the enterprise’s motivation for technological innovation [37]. The norm of the cooperation environment enhances the enterprises’ adherence to the cooperation principle of "risk sharing, mutual benefit, complementary advantages, and common development", and promotes innovative activities. A good cooperation environment plays a positive role in promoting enterprise innovation. It improves the knowledge transfer efficiency of enterprises, reduces the cost of enterprise research and development, establishes a stable cooperative relationship, improves the enthusiasm of enterprises for cooperative research and development, and plays a positive catalytic role in the performance of technological innovation of enterprises.

## 3. Research methods

The qualitative comparative analysis (QCA) method was proposed by Berg-Schlosser, De Meur [38]. It is a research method, and a set of analytical tools that considers the ideas of "configuration comparison" and "set theory". This paper adopts the fsQCA approach for various reasons. First, QCA is "results-driven". It allows the assessment of the "cause and effect of multiple concurrencies" by identifying those different context-specific causal paths leading to the same outcome. Second, previous studies involved regression and interaction analyses of antecedent variables. The QCA method reveals complex relationships between a set of underlying mechanisms, which often do not specify a direct relationship between a single factor and an outcome, but rather illustrate the relationship between a set of factors and their outcomes. It is different from regression, canonical correlation analysis, discriminant analysis and cluster analysis, which regard each factor as an antecedent factor of the result. QCA reveals the influence of complex relationships among multiple antecedent factors on the result. Third, from the perspective of management practice, the enterprise promotes organizational learning and external cooperation, the enterprise still faces low technological innovation performance. However, QCA reveals asymmetry issues. In other words, the QCA method explains the reasons why high-tech innovation performance and non-high-tech innovation performance are not opposite. Fourth, this study selects 20 cases and selects 6 interpretation conditions, which is exactly in line with the QCA analysis of medium cases (10–40 cases).

### 3.1 Ethics statement

This study was conducted in accordance with the Declaration of Helsinki, and Guangxi Normal University reviewed and approved the study protocol. All participants read and signed a consent form before they participated in the study.

### 3.2 Procedure

This study draws on the mature scales of previous studies and modifies and develops new scales according to the current research status. Organizational learning has three dimensions, including commitment learning, common vision, open mindedness, and cooperation intention. External cooperation has 3 dimensions, including cooperation intention, cooperation mode, cooperation environment. The scope of the questionnaires was mainly concentrated in the three provinces of Henan, Fujian and Hubei. Data collection covered as many technologically innovative enterprises as possible by means of questionnaires. This study seeks to ensure sufficient homogeneity and heterogeneity of the case population [39]. There are 20 cases selected, six antecedent variables, and a balance between the number of cases and the number of conditions. In this study, 400 questionnaires were distributed over a period of five months, and 111 invalid questionnaires were excluded. The deletion of invalid questionnaires follows these criteria: first, if respondents selected the same response category for all items; or, second, if the responses to the questionnaire were incomplete. There were 289 valid questionnaires, and the recovery rate was 72.25%. 72% of the survey respondents were managers, including 54% of middle and senior managers. The sample covers businesses of different organizational sizes and is therefore well-represented. This study used SPSS software to verify the results. The basic information of the company is shown in Table 1.

**Table 1:** The basic information of the company.

Company abbreviation	Enterprise nature	Organizational size	Industry	Company age	Average annual sales revenue in the past two years (Chinese yuan)	The ratio of total R&D investment to total sales revenue in the past two years	Company location
HNHY	Private Enterprise	Less than 100 persons	Electronic information	5~10 years	3 million ~10 million	3%~5%	Zhengzhou, Henan
FJXF	State-owned and state-controlled enterprise	100 ~ 500 persons	Electronic information	5~10 years	1 million ~3 million	1%~3%	Fuzhou, Fujian
HNDD	State-owned and state-controlled enterprise	501~1,000 persons	material equipment	More than 20 years	60 million ~100 million	3%~5%	Zhengzhou, Henan
FJLTCM	Private Enterprise	Less than 100 persons	Information service	5~10 years	1 million~3 million	Below 0.5%	Fuzhou, Fujian
WHYS	Private Enterprise	Less than 100 persons	Electronic information	5~10 years	300 million ~ 1 billion	10%~15%	Wuhan, Hubei
ZZZXCH	Private Enterprise	100~500 persons	Electronic information	5~10 years	60 million ~100 million	3%~5%	Zhengzhou, Henan
WHADF	Private Enterprise	501~1,000 Less than 100 persons	Advanced Equipment Manufacturing	5~10 year	3 million~10 million	1%~3%	Wuhan, Hubei
WHTM	State-owned and state-controlled enterprise	More than 1000 persons	Advanced Equipment Manufacturing	11~20 years	More than 5 billion	10%~15%	Wuhan, Hubei
WHFHZZ	State-owned and state-controlled enterprise	501~1,000 persons	Electronic information	5~10 years	300 million ~1 billion	5%~10%	Wuhan, Hubei
ZZXF	State-owned and state-controlled enterprise	100~500 persons	New material preparation	5~10 years	100 million ~300 million	5%~10%	Zhengzhou, Henan
HNTG	State-owned and state-controlled enterprise	More than 1,000 persons	Medical equipment	More than 20 years	100 million ~300 million	3%~5%	Nanyang, Henan
WHSTL	Sino-foreign joint venture	501~1000 persons	Advanced equipment manufacturing	5~10 years	300 million ~1 billion	3%~5%	Wuhan, Hubei
WHJX	Private Enterprise	100~500 persons	Information service	5~10 years	10 million~30 million	Above 15%	Wuhan, Hubei
XMXX	Private Enterprise	Less than 100 persons	Information service	11~20 years	30 million~60 million	3%~5%	Fujian Xiamen
XXHB	Sino-foreign joint venture	100~500persons	Biopharmaceuticals	11~20 years	100 million ~300 million	3%~5%	Xinxiang, Henan
HYKJ	Private Enterprise	Less than 100 persons	Electronic information	5~10 years	3–10 million	3%~5%	Zhengzhou, Henan
KSBJHK	Private Enterprise	Less than 100 persons	material equipment	5~10 years	3–10 million	3%~5%	Zhengzhou, Henan
ZGLX	Private Enterprise	501~1,000 persons	New material preparation	11~20 years	100 million ~300 million	5%~10%	Zhengzhou, Henan
XMSX	Private Enterprise	100~500 persons	Information service	11~20 years	100 million ~300 million	5%~10%	Fujian Xiamen,
ZJSJ	State-owned and state-controlled enterprise	More than 1,000 people	Equipment manufacturing	11~20 years	1 billion ~5 billion	0.5%~1%	Wuhan, Hubei

### 3.3 Measurement

All scales in this study adopted the 7-point Likert scale, ranging from 1 for "completely disagree" to 7 for "completely agree".

1. **Outcome variables.** The scale draws on Zhang and Li [40]. This study starts from two dimensions of technological innovation efficiency and technological innovation benefits. It includes the number of new product development, the number of patent applications, the cycle of new product development, the success rate of new product development, the number of new products developed market share, new product sales and the cost reduction rate. There are a total of seven items. For example, "Our company files more patents each year than our competitors in the same industry".
2. **Antecedent variables.** *Organizational learning*. This paper adopts the scale compiled by Sinkula, Baker [26], which is divided into three dimensions: learning commitment, shared vision and open mind, with a total of 12 items. Among them, there are three items for commitment learning, for example, "Our company sees organizational learning as the foundation for a sustainable future." There are four items of common vision. For example, "All employees, levels and departments of our enterprise have a common goal or vision. There are five items of open mindedness, for example, "Our enterprise managers encourage employees to think from different angles."

*External cooperation*. This paper adopts the scale compiled by Powell, Koput [36] and Bonaccorsi and Piccaluga [41], which has three dimensions of cooperation intention, cooperation mode and cooperation environment, with a total of 13 items. There are four items of cooperation intention, for example, "our enterprise can properly handle the relationship with partners and maintain long-term cooperation". There are four items of cooperation mode, for example, "our enterprise has obtained more market information through external cooperation". There are five items of cooperation environment, for example, “government policies encourage cooperation between enterprises and institutions”.

## 4. Data analysis

### 4.1 Common method bias

In this study, Harman’s single factor test was used to test the homology to effectively avoid homology bias. The results showed ​that the variance explained by the first common factor was 34.22%, which was less than 40% [42]. It can be considered that there is no serious common method bias.

### 4.2 Reliability and validity analysis

Delete the items with standardization factor loading below 0.5 and keep commitment learning 3 items, common vision 3 items, open mindedness 5 items, cooperation intention 4 items, cooperation mode 4 items, cooperation environment 5 items, and technical innovation performance 5 items.

As shown in Table 2, Cronbach’s alpha coefficient of all variables is greater than 0.7. The composite reliability (CR) minimum is 0.793. The results of exploratory factor analysis (EFA) showed that all variables’ KMO value was greater than 0.7, the cumulative explained variance variation was the smallest at 78.11%, the factor loading of all items was greater than 0.6, and the AVE of each variable was greater than 0.5. It shows that all variables have good reliability and validity.

**Table 2:** Reliability and validity analysis.

	Organizational learning			External cooperation			TIP
	CL	CV	OM	CI	CM	CE	
Mean	5.11	5.41	5.51	5.13	5.12	4.26	5.01
SD	0.85	0.97	0.76	0.84	0.87	0.73	0.92
max	7.00	7.00	6.80	7.00	7.00	7.00	7.00
min	1.00	1.33	3.00	1.00	1.25	1.00	1.60
Cronbach’s α	0.788	0.884	0.843	0.854	0.821	0.835	0.809
KMO value	0.705***	0.707***	0.835***	0.811***	0.806***	0.797***	0.835***
CR	0.787	0.878	0.834	0.845	0.823	0.869	0.819
AVE	0.552	0.748	0.521	0.577	0.538	0.578	0.540

Note 1

*** *p*<0.001.

Note 2: CL: Commitment learning; CV: Common vision; OM: Open mindedness; CI: Cooperation intention; CM: Cooperation mode; CE: Cooperation Environment; and TIP: Technological innovation performance

### 4.3 Calibration of variables

Calibration is an important step in QCA research. It is the process of converting cases into collective membership scores. In the calibration process, researchers need to abide by the principles of external standards, rationality and transparency. Therefore, correct calibration can solve the mechanical behaviour of blindly giving qualitative anchor points and abusing descriptive statistics. Moreover, full process disclosure and information interpretation are conducive to readers’ mastery of the "core" of causal mechanism. There are three common methods: direct assignment, direct calibration and indirect calibration. Among them, the direct calibration method is widely used by most researchers because it highlights the characteristics of formalization and the use of statistical models [43]. According to the previous research, combined with the recommended value given by Tosmana software, the intersection point is selected, and the 20% quantile and 80% quantile of the sample data are selected as the critical values of nonfully affiliated and fully affiliated, as shown in Table 3. This paper uses fsQCA 3.0 software for analysis.

**Table 3:** Calibration thresholds for variables.

Variables		Critical value		
		Fully affiliated	Intersection	Non-fully affiliated
Organizational learning	CL	6.00	5.00	4.33
	CV	6.33	5.33	4.67
	OM	6.20	5.60	5.00
External cooperation	CI	5.75	5.25	4.50
	CM	6.00	5.00	4.50
	CE	5.25	4.25	3.50
TIP		5.80	5.00	4.40

Note: CL: Commitment learning; CV: Common vision; OM: Open mindedness; CI: Cooperation intention; CM: Cooperation mode; CE: Cooperation Environment; and TIP: Technological innovation performance

### 4.4 Analysis of necessary conditions

The sufficiency analysis of conditional configuration is carried out separately to make appropriate assumptions about the logical remainder in the process of logical minimization and avoid the trap of taking the conditions that always appear in the results of sufficiency analysis as necessary conditions. The necessary condition is measured according to the consistency score. If the consistency score is greater than or equal to 0.9, it is a necessary condition. On the contrary, it is not a necessary condition. The necessary condition analysis is performed using fsQCA software, as shown in Table 4.

**Table 4:** Necessity test of antecedent conditions.

Antecedent variables		Outcome variables	
		High technological innovation performance	Non-high technological innovation performance
Organizational learning	CL	0.94	0.69
	~CL	0.52	0.81
	CV	0.75	0.78
	~CV	0.63	0.61
	OM	0.83	0.69
	~OM	0.62	0.78
External cooperation	CW	0.84	0.55
	~CW	0.65	0.92
	CM	0.93	0.70
	~CM	0.60	0.86
	CE	0.81	0.65
	~CE	0.65	0.82

Note1: "~" means "non" of logical operation.

Note2: CL: Commitment learning; CV: Common vision; OM: Open mindedness; CI: Cooperation intention; CM: Cooperation mode; CE: Cooperation Environment; and TIP: Technological innovation performance

As shown in Table 4, the analysis results of the necessary conditions for high enterprise technological innovation performance show that the consistency of high commitment learning and the matching cooperation mode is greater than 0.9, and the consistency of other conditions is less than 0.9. This indicates that high commitment learning and fit cooperation mode may be necessary conditions for explaining high enterprise technological innovation performance; in the analysis of the necessary conditions for non-high enterprise technological innovation performance, the consistency of negative cooperation intention is greater than 0.9. This suggests that negative cooperation intention may be a necessary condition to explain the performance of non-high technological innovation performance.

### 4.5 Configuration analysis

The result of the antecedent configuration whose original consistency score is equal to the threshold is assigned as 1, otherwise, it is set as 0. The critical value of PRI consistency is greater than 0.75, and 0.70 is acceptable. We set the PRI consistency value to 0.70 and the original consistency threshold to 0.8. As shown in Table 5, the software derives complex, intermediate, and parsimonious solutions. The intermediate solution has the characteristics of reasonable evidence, moderate complexity and does not allow the elimination of necessary conditions. Intermediate solutions are preferred for the interpretation of results in QCA studies. The expression method refers to the presentation method proposed by Ragin [12] and Fiss, Sharapov [44].

**Table 5:** Configurations of high technological innovation performance.

Antecedent condition	High technological innovation performance				Non-high technological innovation performance			
	H1	H2	H3	H4	NH1	NH2	NH3	NH4
Commitment learning	●	●	●	●	○	○		●
Common vision		●	●	○		●	●	●
Open mindedness	●	●	●	●	●	○	○	○
Cooperation intention	●	●	●	○	○	○	○	○
Cooperation mode	●		●	●	○	○	○	●
Cooperation Environment	○	○		●	○		○	●
Consistency	0.972	0.960	0.977	0.974	0.987	0.998	0.985	0.984
Coverage	0.564	0.434	0.575	0.402	0.482	0.540	0.500	0.437
Unique coverage	0.049	0.004	0.121	0.016	0.118	0.031	0.021	0.041
Solution consistency	0.964				0.975			
Solution coverage	0.729				0.749			

Note: Black circles indicate the presence of a condition, and circles indicate its absence. Large circles represent the core condition. Small circles represent the peripheral condition. Blank spaces indicate “don’t care”.

There are four configurations of high technological innovation performance: H1, H2, H3 and H4. Among them, the consistency of the four configurations is 0.972, 0.960, 0.977, and 0.974, respectively, and their values are all greater than 0.8, indicating that all items are sufficient conditions for high-tech innovation performance. The consistency of the solution is 0.964, which means that 96.40% of the technical innovation performances show a high level in all the cases that meet the four types of conditions. The overall coverage of the solution is 0.729, indicating that the four types of conditional configurations can explain 72.90% of the cases of high enterprise technological innovation performance.

In addition, there are four configurations of non-high-performance innovation: NH1, NH2, NH3 and NH4, and their configuration consistency is 0.987, 0.998, 0.985, and 0.984, respectively. The consistency of the solution is 0.975, which means that 97.50% of the technical innovation performances show a low level in all the cases that meet these four conditions. The overall coverage of the solution is 0.749, proving that this configuration is a sufficient condition for the results, explaining the reason for nearly 74.90% of non-high-tech innovation performance cases.

#### 4.5.1 Model analysis of high technological innovation performance

1. *Consciousness-system synergy type*. Conditional configuration H1: CL *OM *CI *CM *~CE, which indicates that no matter whether the enterprise has a common vision or not, as long as the enterprise has a high level of commitment learning, enough open-mindedness, strong cooperation intention and a suitable cooperation mode. Even in the absence of a stimulating and supportive cooperation environment, high technological innovation performance can still be produced. Among them, commitment learning, cooperation intention and cooperation mode are the core conditions, and open mindedness and negative cooperation environment are the supplementary conditions. Dynamic environmental changes force enterprises to develop the habit of dynamic thinking to cope with fierce market competition. Commitment learning causes synergy and resource integration effect, namely, the collaborative technology learning effect of clusters, which is conducive to the deeper transfer and sharing of knowledge between enterprises and partners. The greater the enterprise commitment learning, the higher the effect of social capital on the enterprise technological innovation performance, and the more patents, which promotes the enterprise technological innovation performance and maintains a competitive advantage. However, the external learning of enterprises has high learning costs. Cooperation intention determines the way and efficiency of organizational members to acquire tacit knowledge. The stronger the cooperation intention, the greater the probability of enterprises acquiring tacit knowledge, which reduces the cost of organizational learning. In addition, the cooperation intention reflects the trust and cooperation of the company to its partners [45], which not only improves the external participation of the company, but also promotes the performance of the company. The choice of a suitable cooperation method represents the company’s accurate understanding and positioning of its own system, improves the sharing and exchange of knowledge resources among members of the organization, stimulates the innovation potential of the company’s employees, and improves the performance of technological innovation.
2. *Consciousness-led type*. Conditional configuration H2: CL *CV *OM *CI *~CE, indicating that no matter whether the enterprise has a suitable cooperation mode, the enterprise has a high commitment learning, the same development vision, and is not limited to the traditional open mindedness and strong cooperation intention. Even without a stimulating and supportive cooperation environment, high technological innovation performance can still be driven. Among them, commitment learning and cooperation intention are the core conditions, and common vision, open mindedness, and negative cooperation environment are marginal conditions. Commitment learning represents the common learning intention of enterprise members. The higher the level of commitment learning, the greater the learning enthusiasm of organizational members, and the lower the probability of cooperation conflicts. Then the frequency of cooperation interaction and cooperation performance are improved, and the greater the enthusiasm of enterprise members to carry out innovation activities, the greater the possibility of successful technological innovation of the enterprise. With the intensification of global competition and the advent of the era of the knowledge economy and digital economy, voluntary cooperation among enterprises is highly respected. Employees trust and learn from others based on the cooperation intention of employees, which improves work efficiency, leads to forming interpersonal networks, improves innovation skills, promotes the circulation, acquisition and absorption of knowledge, and develops and innovates new products or services of enterprises. It positively effects enterprise technological innovation performance [46].
3. *Quasi-comprehensive type*. Conditional configuration H3: CL *CV *OM *CI *CM, which indicates no matter whether the enterprise has a cooperation environment or not, as long as, the enterprise has a commitment learning, common vision, open mindedness, cooperation intention and cooperation mode, then the technological innovation performance of the enterprise can be effectively improved. Among them, commitment learning, cooperation mode and cooperation intention are the core conditions, and common vision and open mindedness are supplementary conditions. Commitment learning drives enterprise innovation [43] and is the core of enterprise technological innovation performance. Development cooperation mainly improves customer satisfaction through product quality and efficiency, helps enterprises fully understand the unpredictable potential market demand, and improves enterprises technology innovation performance. The object of exploratory collaboration is new knowledge, new skills and new products. It improves business performance. Cooperation intention represents the preference and desire of enterprises to cooperate in technological innovation activities. A higher cooperation intention enhances the cooperation relationship between organizations, promotes communication between organizations, and improves technological innovation of enterprises. Enterprises with high-level commitment learning and a strong innovation intention, will have a more active technological innovation.
4. *All-round drive type*. Conditional configuration H4: CL *~CV *OM *~CW *CI *CE, which indicates that even enterprises lack a common vision and strong cooperation intention. However, enterprises with a high degree of commitment to learning, sufficient open-mindedness, suitable cooperation mode, and a stimulating and secure cooperation environment, still promote enterprises technological innovation performance. Among them, commitment learning, cooperation mode, and lack of common vision are the core conditions, and open mindedness, low cooperation intention, and a positive cooperation environment are supplementary conditions. Development cooperation promotes enterprise products and services and points out the direction for enterprise research and development. Exploratory cooperation injects new knowledge and technology into the enterprise, develops the market, and drives the competitive advantage. However, no matter what kind of cooperation method, it improves enterprise technological innovation performance. Common vision helps enterprises to position themselves and establish common goals. It is for employees to establish a sense of protagonism and improve their perception of and insight into the environment. The sharing of a common vision promotes the sharing of employees’ values, makes employees have a sense of ownership, and improves the convenience and effectiveness of adjustment and reconstruction for enterprises. The technological innovation performance of enterprises is affected by organizational resilience, and the organizational learning ability and environmental adaptability of enterprises are closely related to organizational resilience. With the support of a stimulating and supportive cooperation environment, enterprises pay more attention to organizational learning and are willing to make changes. The stronger the organization is, the more resilient it will be under the pressure of the dynamic environment [47].

Comparing the four types of technological innovation modes, the conditional configuration H3 is slightly higher than that of H1, H2 and H4. It explained 57.5% of high technological innovation performance results, covering three cases. The four configurations of high technological innovation performance prove that enterprises jointly drive their technological innovation performance through the synergy between organizational learning and external cooperation. The other four innovation models show that there is not only a single path to stimulate technological innovation of enterprises, and that appropriate organizational learning and external cooperation factors can be linked and matched to improve technological innovation performance of enterprises through multiple paths.

#### 4.5.2 Model analysis of non-high technological innovation performance

Conditional configuration NH1: ~CL *OM *~CI *~CM *~CE, which indicates that no matter whether the enterprise has a common vision or not, as long as the enterprise has insufficient commitment learning, lacks a strong cooperation intention, does not adopt a suitable cooperation mode, and does not actively cooperation environment support. Even if the enterprise is open minded, the technological innovation performance of the enterprise will be inhibited. Among them, low commitment learning and cooperation intention, and wrong cooperation mode are the core conditions, and open mindedness and negative cooperation environment are supplementary conditions.

Conditional configuration NH2: ~CL *CV *~OM *~CI *~CM, which indicates whether the firm has active cooperation environment support or not. As long as the company lacks commitment learning and adopts traditional behaviour, there is no strong cooperation intention and a reasonable cooperation mode. Even if enterprises share a common vision, non-high technological innovation performance will still occur. Among them, low commitment learning, negative cooperation intention and unreasonable cooperation mode are the core conditions, and common vision and non-open mindedness are supplementary conditions.

Conditional configuration NH3: SV *~OM *~CI *~CM *~CE, which indicates whether the firm has a high level of learning commitment. As long as there is a lack of open mindedness, low cooperation intention, unreasonable cooperation mode and a negative cooperation environment. Even if companies share a common vision, their technological innovation performance will still be inhibited. Among them, low-level cooperation intention and unsuitable cooperation mode are the core conditions, and common vision, lack of open mindedness and lack of cooperation environment support are supplementary conditions.

Conditional configuration NH4: CL *SV *~OM *~CI *CM *CE, which shows that although the enterprise has commitment learning, the common vision, adopts a reasonable cooperation mode and has positive cooperation environment support. However, as long as the enterprise lacks open mindedness and cooperation intention, the technological innovation performance of the enterprise is still ineffective. Among them, commitment learning, low level of open mindedness and negative cooperation intention are the core conditions, and common vision, cooperation mode and cooperation environment are supplementary conditions.

Comparing the above 8 configurations, it is found that the factors that affect the performance of technological innovation of enterprises are asymmetric, that is, the four types of high technological innovation performance models are not the opposite of non-high technological innovation performance models. Comparing H1 and H2, it is also found that there is a substitution effect of high common vision and high cooperation mode, that is, under the premise of high commitment learning, high open mindedness, high cooperation intention and lack of high cooperation environment, the enterprise has high common vision or high cooperation mode. Both approaches can lead to high enterprise technological innovation performance.

### 4.6 Robustness test

Researchers are interested in the sufficiency configuration of the results. Therefore, a robustness test is usually an analysis of the sufficiency configuration. Robustness checks should be performed in preference to set theory-specific methods [48], such as adjusting calibration thresholds, changing case frequencies, changing consistency values, or adding other conditions relevant to the results. Some scholars combine the econometric method [49] with the QCA method and adopt different measurement methods to test the robustness.

We conduct robustness tests by adjusting the consistency threshold and changing the calibration threshold. First, the original consistency threshold was raised from 0.80 to 0.90, the case frequency remained the same, and the adjusted results did not change. Second, the complete membership threshold of all variables was changed to the 90% quantile, the completely non-membership threshold was changed to the 10% quantile [50], the original consistency threshold was 0.90, and the case frequency remained unchanged. The results are shown in Table 6. shown. Comparing Tables 5 and 6, it can be seen that the conditions of configuration H1, H2, H3, and H4 are exactly the same. In terms of fitting parameters, the agreement of the four configurations, the overall agreement of the solution, and the overall coverage of the solution have only minor changes. Therefore, the conclusions of this study have good robustness.

**Table 6:** Configuration of high technological innovation performance.

Antecedent condition	High technological innovation performance			
	H1	H2	H3	H4
Commitment learning	●	●	●	●
Common vision		●	●	○
Open mindedness	●	●	●	●
Cooperation intention	●	●	●	○
Cooperation mode	●		●	●
Cooperation Environment	○	○		●
Consistency	0.982	0.986	0.983	0.975
Coverage	0.662	0.600	0.706	0.596
Unique coverage	0.057	0.003	0.098	0.025
Solution consistency	0.972			
Solution coverage	0.769			

Note: Black circles indicate the presence of a condition, and circles indicate its absence. Large circles represent the core condition. Small circles represent the peripheral condition. Blank spaces indicate “don’t care”.

## 5. Discussion

### 5.1 Theoretical contributions

Enterprise technological innovation is a very complex process, and new ventures face a more dynamic and complex business environment. Therefore, the technological innovation process faces many more complex problems. The theoretical contributions of this study are as follows.

First, previous studies have confirmed that corporate social responsibility and social capital have a positive impact on technological innovation performance [51]. This study enriches research on technological innovation performance and reveals the interaction mechanism between organizational learning and external cooperation. The effect of configuration synergy between different elements on the technological innovation performance of new ventures by adopting the method of qualitative comparative analysis of fuzzy sets, we found that there is a substitution effect between elements, and commitment learning, common vision, open mindedness, cooperation intention, cooperation mode and the cooperation environment always accompany the technological innovation process of new ventures. It provides theoretical support and practical guidance for enterprises to use organizational learning and external cooperation in the environment of innovation-driven economic development and also has reference significance for other scholars to conduct related research.

Second, this paper studies technological innovation performance from the perspective of organizational learning and external cooperation and explores the configuration that effects the high-tech and low-tech innovation performance of enterprises. Previous research on technological innovation performance was mostly used for corporate governance [52], and this paper explains the important role of organizational learning and external cooperation on technological innovation.

Third, this paper finds that the configuration of organizational learning and external cooperation promotes enterprise technological innovation based on the theoretical logic of “organizational learning-external cooperation-technological innovation”. It is no longer limited to the impact of organizational learning on technological innovation [53, 54]. Rather, it explains its effect mechanism on enterprise technological innovation from the holistic perspectives of learning (commitment learning, common vision, and open-mindedness) and cooperation (cooperation intention, cooperation mode, and cooperation environment). In addition, it found that the path affecting the performance of technological innovation of enterprises is asymmetric, that is, the way to generate high-technological innovation performance is not completely opposed to the path for generating non-high technological innovation performance, which makes up for the lack of traditional regression research that is difficult to apply to real management practices.

### 5.2 Practical contributions

First, we realize that there are multiple concurrent and complex effects of organizational learning factors and external cooperation factors, and, thus, pay attention to the promotion of organizational learning and external cooperation on the performance of enterprise technological innovation. In other words, enterprises of different types and sizes face different environments, and their willingness to carry out organizational learning and external cooperation varies. Enterprises should have a sense of the overall situation, adopt different cooperation methods according to their own conditions from an overall perspective, and always maintain positive learning behaviour in a dynamic environment. On the one hand, learning and cooperation interact to encourage enterprises to effectively explore new resources and obtain more heterogeneous information conducive to technological innovation. On the other hand, enterprises improve the sensitivity and flexibility of resource acquisition through learning and cooperation, and achieve a good cooperative relationship between enterprises to speed up the absorption and utilization of knowledge, respond positively in the face of complex and dynamic external environment, and effectively improve the performance of enterprise technological innovation.

Second, enterprises should constantly improve their learning commitment, willingness to cooperate and the ability to cooperate to realize long-term technological innovation. The research shows that high learning commitment, cooperation intention and matching cooperation mode play important roles in promoting enterprise technological innovation. As the core condition of high technological innovation performance, commitment learning appears in all four paths. As the core condition of high technological innovation performance, cooperation intention and cooperation mode appear in three paths, highlighting their importance in the process of enterprise technological innovation. Enterprises can enhance their members’ access to heterogeneous knowledge resources and information resources and enhance their confidence in technology R&D and innovation and change by improving their learning commitment and willingness to cooperate. Choosing a suitable mode of cooperation is conducive to establishing a knowledge-sharing mechanism with the outside world, promoting the transformation of external knowledge and information into internal resources, strengthening the efficiency of organizational learning and external cooperation, and finally realizing technological innovation.

Third, enterprises should improve their commitment to learning, cooperation intentions and cooperation methods to achieve long-term technological innovation. This study shows that commitment learning, cooperation intention, and suitable cooperation mode play important roles in promoting enterprises technological innovation. As the core condition of high technological innovation performance, learning commitment appears in all four paths. As the core conditions of high technological innovation performance, cooperation intention and cooperation mode appear in the three paths, which highlights their importance in the process of enterprise technological innovation. Enterprises enhance the confidence of technological research and development and innovation and change and enhance organizational members’ access to heterogeneous knowledge resources and information resources by improving the organization’s commitment learning and cooperation intention. Enterprises choose appropriate cooperation methods to promote the establishment of knowledge sharing mechanism with the outside world, promote the transformation of external knowledge and information into internal resources, strengthen the efficiency of organizational learning and external cooperation, and ultimately achieve technological innovation.

Finally, the occurrence of technological innovation performance of high-tech firms and non-high-tech firms is asymmetric. And the occurrence of high enterprise technological innovation performance is also asymmetric. Therefore, managers cannot simply identify the opposite of the factors that produce low technological innovation performance as a condition for producing high technological innovation performance. Enterprises need to strengthen their own organizational learning ability, have the courage to carry out learning behaviours, actively carry out external cooperation and speed up the dissemination and recreation of knowledge. Managers must accurately position the enterprise, realize the role of core conditions, and recognize the advantages and disadvantages of the enterprise to match and choose the correct path to improve the performance of technological innovation of the enterprise according to changing and complex environmental changes.

### 5.3 Research limitations and suggestions

This study has the following limitations.

First, it does not divide the growth stages of the case enterprises, nor does it take into account the different attitudes towards organizational learning and external cooperation in different growth stages of enterprises. The degree of emphasis on technological innovation of enterprises is also different, and enterprises adopt different learning methods and cooperation methods at different growth stages. Then, the configuration effects of organizational learning factors and external cooperation factors may vary due to the different growth stages of enterprises. Therefore, future research should explore the differences of technological innovation models from the growth stage of enterprises and consider using the dynamic QCA method to conduct related research in the time dimension.

Second, the research object of this paper is only 20 enterprises, and the sample size is not large. Therefore, further in-depth research is needed to verify the generalizability of the research conclusions. This study did not conduct more in-depth field research on typical enterprises or representative enterprises. Instead, we use the data obtained from the questionnaire to judge the impact of organizational learning and external cooperation on the technological innovation of enterprises. There is a lack of in-depth analysis of existing technological innovation models. Specific case companies should be studied in combination with grounded theory in future research.

Finally, this study is limited to the effects of organizational learning and external cooperation factors on technological innovation performance. Future research should focus on exploring the differences in technological innovation performance of enterprises in different industries or different scales under different cooperation models and organizational learning methods.

## 6. Conclusion

This study surveyed 20 companies in Henan, Fujian and Hubei provinces. From the perspective of organizational learning and external cooperation, the fsQCA research method and configuration are used to integrate six conditional factors at two levels of organizational learning and external cooperation, and to explore the multiple concurrent and causal complex mechanisms that affect enterprise technological innovation performance. The results of this study are as follows. First, neither organizational learning nor external cooperation factors alone promote enterprise technological innovation, thereby improving enterprise technological innovation performance. Second, there are four types of configurations of high technological innovation performance.: (1) High commitment learning, high open mindedness, high cooperation intention, high cooperation mode and lack of high cooperation environment linkage and matching; (2) High commitment learning, high common vision, high open mindedness, high cooperation intention and lack of high cooperation environment linkage and matching; (3) High learning commitment, high common vision, high open mindedness, high cooperation intention and high cooperation mode linkage and matching; and (4) High commitment learning, lack of common vision, high open mindedness, lack of high cooperation intention, high cooperation mode and lack of cooperation environment linkage matching. Of these configurations, the third type of conditional configuration is more effective in improving enterprise technological innovation performance. Finally, there are four types of conditional configurations that inhibit enterprise technological innovation, and they have an asymmetric relationship with the four types of configurations that affect the high enterprise technological innovation.
